# Exposure to air pollution and its effect on ischemic strokes (EP-PARTICLES study)

**DOI:** 10.1038/s41598-022-21585-7

**Published:** 2022-10-13

**Authors:** Łukasz Kuźma, Sylwia Roszkowska, Michał Święczkowski, Emil Julian Dąbrowski, Anna Kurasz, Wojciech Wańha, Hanna Bachórzewska-Gajewska, Sławomir Dobrzycki

**Affiliations:** 1 Department of Invasive Cardiology, Medical University of Bialystok, Białystok, Poland; 2 Faculty of Economics and Sociology, University of Lodz, Łódź, Poland; 3 Faculty of Management, University of Warsaw, Warsaw, Poland; 4 Department of Cardiology and Structural Heart Diseases, Medical University of Silesia, Katowice, Poland; 5 Department of Invasive Cardiology, Department of Clinical Medicine, Medical University of Bialystok, Białystok, Poland

**Keywords:** Environmental impact, Stroke

## Abstract

It is well known that exceeded levels of particulate matter in the air and other air pollutants harmfully affect the cardiovascular system. Empirical analyses of the effects of these factors on stroke incidence and mortality are still limited. The main objective of our analyses was to determine the association between short-term exposure to air pollutants and stroke incidence in non-industrial areas, more specifically in north-eastern Poland. To achieve this aim, we used data from the National Health Fund on patients hospitalized for stroke between 2011 and 2020 in the largest city of the region described as the Green Lungs of Poland. The pollution levels and atmospheric conditions data were obtained from the Provincial Inspectorate for Environmental Protection and the Institute of Meteorology and Water Management. Using daily data on hospitalizations, atmospheric conditions, and pollution, as well as ordered logistic regression models the hypotheses on the impact of weather and air pollution conditions on ischemic strokes were tested. The study group included 4838 patients, 45.6% of whom were male; the average patient age was approximately 74 years. The average concentrations of PM_2.5_ were 19.09 µg/m^3^, PM_10_ 26.66 µg/m^3^ and CO 0.35 µg/m^3^. Analyses showed that an increase in PM_2.5_ and PM_10_ concentrations by 10 µg/m^3^ was associated with an increase in the incidence of stroke on the day of exposure (OR = 1.075, 95% CI 0.999–1.157, P = 0.053; OR = 1.056, 95% CI 1.004–1.110, P = 0.035) and the effect was even several times greater on the occurrence of a stroke event in general (PM_2.5_: OR = 1.120, 95% CI 1.013–1.237, P = 0.026; PM_10_: OR = 1.103, 95% CI 1.028–1.182, P = 0.006). Furthermore, a short-term (up to 3 days) effect of CO on stroke incidence was observed in the study area. An increase of 1 μg/m^3^ CO was associated with a lower incidence of stroke 2 days after the exposure (OR = 0.976, 95% CI 0.953–0.998, P = 0.037) and a higher incidence 3 days after the exposure (OR = 1.026, 95% CI 1.004–1.049, P = 0.022).

## Introduction

Following the latest WHO reports, the number of deaths caused by strokes has emerged since the year 2000 and in 2019 they were responsible for over 6 million deaths. Interestingly, dividing strokes into hemorrhagic and ischemic, a more significant increase was noted in the latter^1^. Although from 1990 to 2019 the contribution of ambient particulate matter with a diameter of < 2.5 μm (PM_2.5_) pollution decreased from 32.5 to 20.1%, in 2019 air quality was the fourth leading risk factor contributing to stroke death and disability combined (DALYs)^2^. Regardless of the fact the exposure to air pollution gradually decreases in high-income countries, the problem is still relevant in Poland—a country with the highest particulate matter with a diameter of < 10 μm (PM_10_) and PM_2.5_ concentrations in the European Union (EU)^3,4^. Particulate matter was the sixth leading risk factor for all-cause mortality in Poland in 2019^3^. Although according to the World Bank, Poland is considered a high-income country, it still has to face post-transformation issues such as a high share of old cars^5,6^. Alleged pathomechanisms of detrimental effects of air pollution include endothelial dysfunction, generation of reactive oxygen species (ROS), and induction of systemic inflammation^7–9^.

The incidence rates of strokes differ between seasons and days of the week. Many studies have reported that stroke incidence rates are the highest in winter and the lowest in summer^10,11^. Possible explanations might include the activation of the sympathetic nervous system during cold seasons, atrial fibrillation paroxysms in winter, and, finally, higher concentrations of air pollutants during heating seasons^12–14^. When analyzing weekly patterns of stroke occurrence, most studies are consistent with a phenomenon of Monday excess^15–17^. It comes in line with other research reporting more frequent occurrences of acute cardiovascular events such as myocardial infarction, aortic dissection, and takotsubo-syndrome on Mondays^16,18,19^. Nevertheless, the mechanisms and underlying factors remain blurred. Besides physiological mechanisms including Monday morning surge in blood pressure and increased double product, some hypotheses include differences in the socioeconomic status of patients^17,20–22^.

Seasonal variations are inseparably connected with changing weather conditions. Many studies provided strong evidence of an association between stroke occurrence and ambient temperature^23–25^. Interestingly, it seems that change in temperature is more important than absolute temperature^25^. The detrimental influence of air pressure and humidity is controversial. Few studies granted evidence on the correlation between daily barometric pressure variation and daily stroke hospitalization, however, Cao et al. (2016) in their meta-analysis found no such relationship^26–28^.

Considering the aforementioned inconsistencies, gaps in evidence, and lack of analysis from this part of Europe, there is a need for further research. The purpose of this study was to investigate temporal variations of stroke incidence in north-eastern Poland and its link with the short-term effect of air pollution in the years 2011–2020.

## Materials and methods

### Study population and data collection

Data on stroke occurrence in the years 2011–2020 were collected from the National Health Fund. The data on air pollutants and weather conditions were obtained from Voivodeship Inspectorate for Environmental Protection in Bialystok and the Institute of Meteorology and Water Management. According to codes in the International Classification of Diseases-10th Revision, we extracted the data for ischemic stroke (ICD-10 I63). Patients were hospitalized in 5 hospitals in or nearby the city: University Clinical Hospital of Bialystok, Voivodeship Hospital in Bialystok, Hospital in Choroszcz, Hospital of the Ministry of Interior and Administration in Bialystok, and the City Hospital in Bialystok. The former three hospitals were responsible for over 99% of admissions. We included only Bialystok adult residents (> 18 years old) admitted to the hospital with ischemic stroke diagnosis (ICD-10 I63) in the years 2011–2020. We excluded hospitalizations of patients transferred from another hospital and hospitalizations during a period without data on air pollutants concentration. We used the concentration of air pollutants (particulate matter with a diameter of 2.5 μm or less (PM_2.5_), 10 μm or less (PM_10_), nitrogen dioxide (NO_2_), sulfur dioxide (SO_2_), carbon monoxide (CO), temperature, mean atmospheric pressure, and relative humidity. In the analysis, the exceedance of air pollution norms was determined based on the World Health Organization (WHO) guidelines concerning air quality. The 24-h concentrations recommended by the WHO are 45 µg/m^3^, 15 µg/m^3^, 40 µg/m^3^, 25 µg/m^3^, 4 mg/m^3^ for PM_10_, PM_2.5_, SO_2_, NO_2_, CO, respectively^29^. Days with missing data were excluded from the analysis.

The study protocol conformed to the ethical guidelines of the 1975 Declaration of Helsinki, and was approved by the Bioethics Committee of the Medical University of Białystok (approval number APK.002.81.2022). Additionally, it was registered in the database of clinical studies www.clinicaltrials.gov (accessed on 5 September 2022, identification number NCT05198492).

### Study region

Białystok is the capital of Podlaskie Voivodeship, a region located in north-eastern Poland. It is inhabited by nearly 300,000 citizens, which makes it the 10th most populated city in Poland and the 2nd considering the population density. Close location to the four national parks, over 18% of the city area covered by forests, and the lack of heavy industry have an impact on the way the city is being perceived^30^. It is often referred to as the capital of the region known as the Green Lungs of Poland and is associated with the resistance to accepting the rush of modern lifestyle. In 2020 men accounted for 46.91% of the city’s population, which resulted in a femininity ratio of 113, and 17.8% of citizens were over 65 years old^30^. With almost 80% of the share, area sources were the main sources of PM_10_ in the studied region in 2018^31^.

### Statistical analysis

Due to the discrete nature of stroke incidences, discrete outcome models (such as logit and probit models) are usually used to link stroke hospital admissions to the observed risk factors^31^. As the daily number of stroke incidences is greater than two, the ordered nature between these levels is its inherent characteristic. Ordered outcome models have a potential advantage over unordered outcome models because they can account for the correlation among neighboring admissions levels by recognizing the ordered nature [for discussion or example see^32,33^. The assumptions of the ordered logit models were rigorously tested using the Wolfe-Gould test^34^ and the test results indicate that the proportional odds assumption has not been violated^35^.

Multivariate analysis was done using a generalized ordered logit model with the number of stroke incidences. The proportional odds ratios were estimated across the categories of the outcome variable—admissions for ischemic strokes in Białystok in 2011–2020. The exposure variables were each air pollutant at a lag of 0–7 days prior to admission. Also, the robustness analysis was performed allowing up to 30-day lags. Additionally, the logit model parameters were estimated for the robustness analyses, and the day on which at least one stroke incidence occurred was taken as the event.

The set of other explanatory variables included temperature, air pressure, relative humidity, day of the week, season (Spring, Summer, Autumn, Winter), year, and share of elderly (women aged over 60 and men over 65 years as a percentage of the total population). The weather variables were incorporated as a natural cubic spline with 4 df (3 equally-spaced knots), to control potential non-linear confounding effects^36,37^. Additionally, to avoid long-term bias we considered the time trend variable and indicator variables for the day of the week to account for intra-weekly variations in stroke incidences. In estimated equations, lag distributions for individual variables were included to get leveling off effect for seasonal and long-term trends^38,39^.

Results are presented as odds ratios (OR) and 95% confidence intervals (95% CI) in association with increases in exposures (per 10 μg/m^3^ increase in PMs and 1 mg/m^3^ in case of others). To control sample heterogeneity the estimates have been conducted in subsamples for the season, (age (65+ and 75+) and gender. All analyses were performed using MS Excel (Microsoft, 2020, version 16.40, Redmond, WA, USA) and Stata Statistical Software, (StataCorp, 2022, version 17, TX, USA).

## Results

From 2010 to 2020 in Białystok, we recorded 4,838 ischemic stroke cases with a daily mean of 1.3 cases [Standard deviation (SD) = 1.2]. Out of the analyzed group, 45.6% of patients were male with a mean age of 74.3 years (SD = 12.2). The standardized morbidity rate (SMR) was 174.6 per 100,000 population/year and the crude morbidity rate was 162.9 per 100,000 population/year. In-hospital deaths accounted for 16.8% (N = 813) (Table 1).

**Table 1:** Ischemic stroke morbidity in Bialystok in the years 2010–2020.

	Ischemic stroke
Total, N	4838
CMR, (100,000 population/year)	162.9
SMR, (100,000 population/year)	174.6
Mean age, (SD)	74.3 (12.2)
Male, % (N)	45.6 (2207)
Daily mean (SD)	1.3 (1.2)
Daily median (minimum – maximum)	1 (0–8)
In-hospital mortality; %, (N)	16.8, (813)

CMR, crude morbidity rate per 100,000 population per year; SD, standard deviation; SMR, standardized morbidity rate per 100,000 population per year.

Taking into account the seasonal distribution of ischemic stroke incidence we observed that the highest number of ischemic stroke cases occurred in the winter season 26.52% (N = 1238), and the lowest in spring 24.78% (N = 1199,), P < 0.001. Regarding the incidence of ischemic strokes throughout the week, the highest number of strokes occurred on Mondays 16.68% (N = 807) vs. Saturdays [12.05% (N = 583)] and Sundays [13.29% (N = 653)], P < 0.001 for pairwise comparsion, (Tables 2 and 3).

**Table 2:** Seasonal variation in the frequency of ischemic stroke in the study population.

Season	Ischemic stroke cases		
	N	%	P
Winter	1283	26.52	< 0.001
Spring	1199	24.78	
Summer	1138	23.52	
Autumn	1218	25.17	

Spring—months from March to the end of May, Summer—June to the end of August, Autumn—September to the end of November, Winter—December to the end of February.

**Table 3:** Weekly changes in the frequency of ischemic stroke occurrence in the study population.

Day of the week	Monday	Tuesday	Wednesday	Thursday	Friday	Saturday	Sunday	P
Ischemic stroke cases, N (%)	807 (16.68)	677 (13.99)	761 (15.73)	675 (13.95)	692 (14.30)	583 (12.05)	653 (13.29)	< 0.001
**Dunn's pairwise comparison of stroke by day**								
Tuesday	0.051							
Wednesday	0.8	0.11						
Thursday	0.03	0.64	0.72					
Friday	0.062	0.45	0.13	0.74				
Saturday	< 0.001	0.049	< 0.001	0.08	0.04			
Sunday	< 0.001	0.45	0.003	0.53	0.47	0.45		

N; number.

The limit of the daily mean given by the WHO guidelines for PM_2.5_ was exceeded on 48% of days, 11.2% days for PM_10,_ and 5.5% days for NO_2_. The daily mean concentrations of PM_2.5_, PM_10_, NO_2_, and SO_2_, were 19.1 µg/m^3^ (SD = 14.5, IQR = 14.3), 26.7 µg/m^3^ (SD = 17.7, IQR = 16.7), 13.9 µg/m^3^ (SD = 6.2, IQR = 7.6), and 3.0 µg/m^3^ (SD = 2.7, IQR = 2.8), respectively. The daily mean for SO_2_ was not exceeded on any day during the observation time (Table 4). The frequency of hospitalizations due to ischemic stroke on the days with exceeded daily norms of PM_2.5_ (1.37/day, N = 1661 vs. 1.26/day, N = 1827; P = 0.04) and NO_2_ (1.5/day, N = 197 vs. 1.31/day, N = 3397; P = 0.02) was significantly higher than on days without exceeded limit values (Table 5).

**Table 4:** Statistics for daily concentrations of air pollutants and weather conditions in the period of 2011–2020.

		NO_2_ µg/m^3^	SO_2_ µg/m^3^	PM_2.5_ µg/m^3^	PM_10_ µg/m^3^	Temp. °C	RH %	Atm. P. hPa
All seasons	No. of daily observations, % (N)	98.4 (3594)	97.8 (3572)	95.5 (3488)	96.1 (3510)	100 (3653)	100 (3653)	100 (3653)
	Daily mean (SD)	13.9 (6.2)	3.0 (2.7)	19.1 (14.5)	26.7 (17.7)	8.1 (8.6)	80.1 (12.4)	1016.1 (8.6)
	Daily 1st quartile	9.5	1.2	9.6	15.5	1.8	71.8	1010.8
	Daily median	12.7	2.4	14.5	22.1	8	81.9	1015.9
	Daily 3rd quartile	17.1	4	24	32.2	15.2	90	1021.6
	Daily IQR	7.6	2.8	14.3	16.7	13.4	18.2	10.8
	Exceeded daily mean (WHO 2021 guidelines values), % (N)	5.5 (197)	0 (0)	48 (1661)	11.2 (393)	N/A	N/A	N/A
Autumn	Daily mean (SD	15.08 (6.2)	5.4 (8.2)	30.87 (18.9)	22.39 (15.4)	3.4 (5.4)	89.2 (7.8)	1017.25 (9.7)
	Daily 1st quartile	10.46	1.58	17.43	11.83	0.1	85.6	1011
	Daily median	14.33	3.27	27.4	19.46	3.6	90.5	1017.55
	Daily 3rd quartile	18.8	5.33	38.86	30.71	7.1	94.8	1024.4
	Daily IQR	8.34	3.75	21.43	18.88	7	9.2	13.4
Spring	Daily mean (SD	12.59 (5)	2.37 (1.5)	21.59 (9.8)	13.01 (6.3)	12.93 (5.4)	70.27 (11.8)	1014.88 (6.5)
	Daily 1st quartile	9.13	1.19	14.76	8.6	9	61.8	1010.5
	Daily median	11.73	2.08	19.84	11.38	13.35	69.8	1014.7
	Daily 3rd quartile	15.23	3.28	25.94	15.8	16.9	79	1019.5
	Daily IQR	6.1	2.09	11.18	7.2	7.9	17.2	9
Summer	Daily mean (SD	12.61 (5.2)	3.41 (7.7)	20.72 (9.1)	10.4 (5.4)	16.54 (3.9)	78.36 (9.2)	1015.56 (5.8)
	Daily 1st quartile	8.92	1.11	14.06	6.67	13.9	72.45	1011.8
	Daily median	11.61	1.9	19	9.5	16.5	78.4	1015.5
	Daily 3rd quartile	15.16	2.95	25.46	13.33	19.3	84.8	1019.15
	Daily IQR	6.25	1.85	11.4	6.66	5.4	12.35	7.35
Winter	Daily mean (SD	15.69 (7.5)	4.65 (3.7)	33.54 (24.3)	28.57 (19)	− 0.54 (5.6)	81.91 (12.1)	1016.67 (11.2)
	Daily 1st quartile	10.43	2.24	17.61	15.13	− 3	74.85	1008.9
	Daily median	14.2	3.93	26.73	23.67	0.5	84.5	1017
	Daily 3rd quartile	19.41	5.9	41.82	36.13	3.2	90.95	1024.4
	Daily IQR	8.98	3.67	24.21	21.01	6.2	16.1	15.5

Atm. P., atmospheric pressure; IQR, interquartile range; N/A, not applicable; N/D, no data; NO_2_, nitrogen dioxide; PM_2.5_, particulate matter with a diameter of 2.5 μm or less; PM_10_ particulate matter with a diameter of 10 μm or less; RH, relative humidity; SD, standard deviation; SO_2_, sulfur dioxide; Temp., temperature; WHO, World Health Organization.

**Table 5:** The comparison of the frequency of hospitalizations due to ischemic stroke on the days with and without exceeded daily norms recommended by the World Health Organization for nitrogen dioxide, particulate matter with a diameter of 10 μm or less, and particulate matter with a diameter of 2.5 μm or less.

Ischemic stroke	Days with exceeded daily limit values for NO_2_ (N = 197)	Days without exceeded daily limit values for NO_2_ (N = 3397)	P
Daily mean (SD), Median (1Q–3Q)	1.5 (1.3) 1 (1–2)	1.31 (1.2) 1 (0–2)	0.02

Ischemic stroke	Days with exceeded daily limit values for PM_2.5_ (N = 1661)	Days without exceeded daily limit values for PM_2.5_ (N = 1827)	P
Daily mean (SD), Median (1Q–3Q)	1.37 (1.2) 1 (0–2)	1.26 (1.2) 1 (0–2)	0.04

Ischemic stroke	Days with exceeded daily limit values for PM_10_ (N = 393)	Days without exceeded daily limit values for PM_10_ (N = 3117)	P
Daily mean (SD), Median (1Q–3Q)	1.45 (1.2) 1 (1–2)	1.31 (1.2) 1 (0–2)	0.6

NO_2_, nitrogen dioxide; PM_2.5_, particulate matter with a diameter of 2.5 μm or less; PM_10_ particulate matter with a diameter of 10 μm or less; SD, standard deviation.

**Figure 1:**
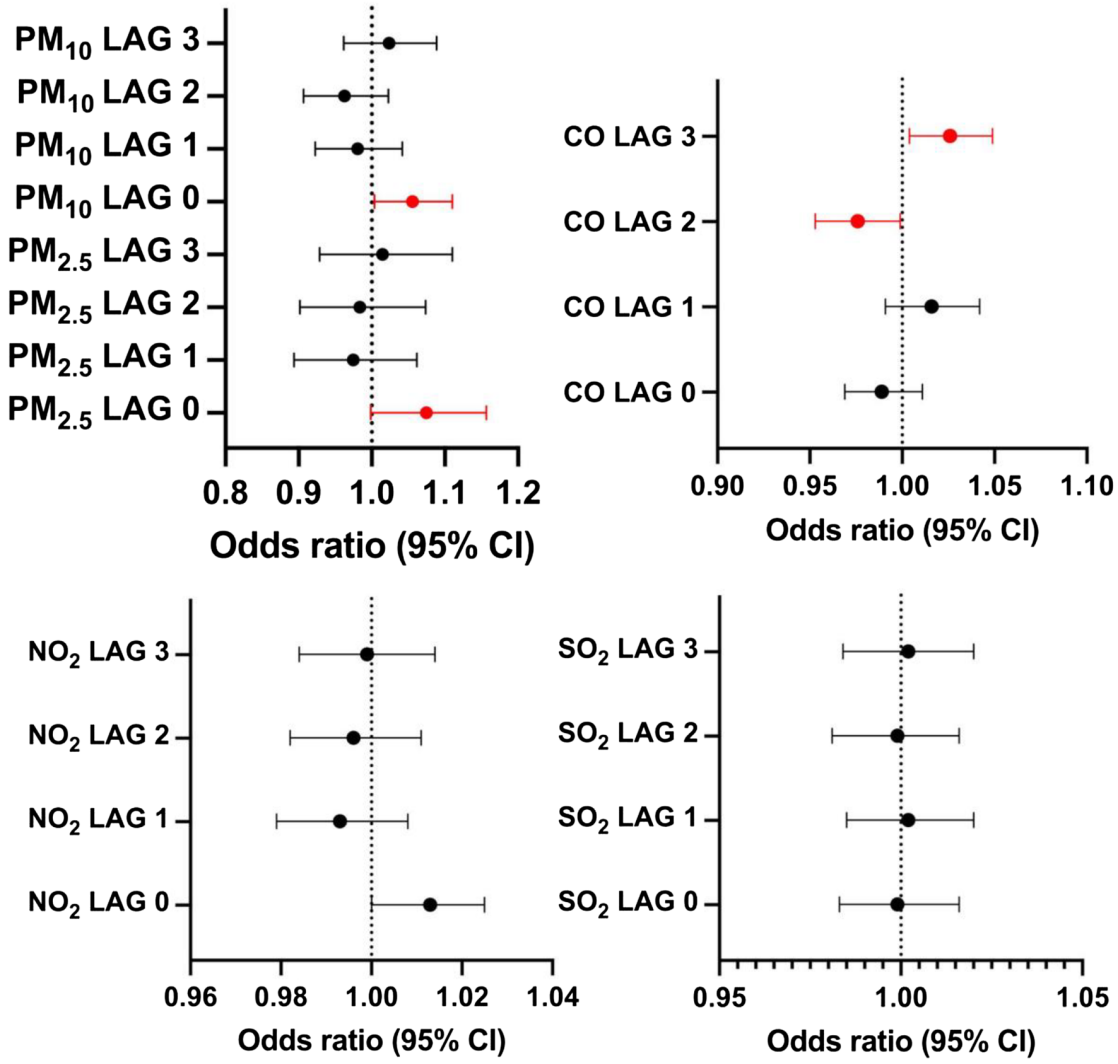
Overall associations between the exposure to short-term pollutants and ischemic stroke incidence.

The effect of air pollution on ischemic stroke incidence was noted on lag 0 for PM_2.5_ with OR = 1.075 (95% CI 0.999–1.157, P = 0.053) as well as for PM_10_ with OR = 1.056 (95% CI 1.004–1.11, P = 0.04). Additionally, the effect of CO on lag 2 was associated with decreased number of strokes OR = 0.976 (95% CI 0.953–0.999, P = 0.04), whereas on lag 3 with increased stroke incidence OR = 1.026 (95% CI 1.004–1.049, P = 0.02) (Fig. 1).

The increase in concentration of PM_2.5_ (1.109, 95% CI 1.002–1.227, P = 0.04), PM_10_ (1.089, 95% CI 1.010–1.174, P = 0.03) and NO_2_ (1.028, 95% CI 1.006–1.050, P = 0.01) in winter season at lag 0 resulted in an increase in ischemic stroke incidence. This effect was also observed for CO in the summer season on lag 3 (OR = 1.037, 95% CI 1.008–1.067, P = 0.01). However, the opposite relation was noted for CO on lag 2 (OR = 0.957, 95% CI 0.924–0.990, P = 0.012) (Table 6).

**Table 6:** Associations between the exposure to short-term pollutants and ischemic stroke incidence over the seasons.

Variables		Winter		Spring		Summer		Autumn	
		OR (95% CI)	P- value	OR (95% CI)	P- value	OR (95% CI)	P- value	OR (95% CI)	P- value
PM_2.5_	LAG 0	1.109 (1.002–1.227)	0.045	1.091 (0.775–1.537)	0.617	1.218 (0.789–1.881)	0.373	1.055 (0.913–1.219)	0.467
	LAG 1	0.907 (0.807–1.021)	0.105	1.332 (0.934–1.900)	0.113	0.631 (0.389–1.024)	0.062	1.006 (0.852–1.189)	0.941
	LAG 2	1.019 (0.905–1.148)	0.755	0.652 (0.456–0.933)	0.019	0.863 (0.527–1.413)	0.558	1.008 (0.848–1.199)	0.924
	LAG 3	1.026 (0.906–1.163)	0.681	1.125 (0.821–1.541)	0.464	0.785 (0.476–1.295)	0.343	0.964 (0.812–1.145)	0.677
PM_10_	LAG 0	1.089 (1.010–1.174)	0.026	1.094 (0.880–1.360)	0.420	1.020 (0.801–1.298)	0.873	1.041 (0.951–1.139)	0.381
	LAG 1	0.916 (0.836–1.004)	0.060	1.056 (0.843–1.322)	0.635	1.055 (0.799–1.392)	0.706	1.029 (0.927–1.141)	0.381
	LAG 2	1.048 (0.957–1.148)	0.309	0.818 (0.655–1.022)	0.077	0.839 (0.635–1.110)	0.219	1.029 (0.927–1.141)	0.595
	LAG 3	0.983 (0.893–1.081)	0.720	0.995 (0.804–1.233)	0.966	1.006 (0.750–1.348)	0.971	1.038 (0.932–1.156)	0.492
CO	LAG 0	2.303 (0.806–6.578)	0.119	0.908 (0.078–10.632)	0.939	0.995 (0.965–1.026)	0.741	0.970 (0.935–1.005)	0.096
	LAG 1	0.452 (0.126–1.616)	0.222	2.510 (0.150–42.033)	0.522	1.019 (0.981–1.059)	0.331	1.015 (0.976–1.057)	0.448
	LAG 2	1.118 (0.313–4.000)	0.864	0.141 (0.008–2.410)	0.176	0.957 (0.924–0.990)	0.012	0.997 (0.959–1.037)	0.885
	LAG 3	0.714 (0.187–2.724)	0.622	0.319 (0.021–4.830)	0.410	1.037 (1.008–1.067)	0.013	1.019 (0.979–1.061)	0.351
NO_2_	LAG 0	1.028 (1.006–1.050)	0.013	0.998 (0.967–1.030)	0.897	1.016 (0.984–1.049)	0.330	0.838 (0.980–1.036)	0.611
	LAG 1	0.975 (0.951–1.000)	0.047	1.008 (0.976–1.042)	0.619	0.987 (0.951–1.025)	0.494	1.004 (0.973–1.037)	0.786
	LAG 2	1.012 (0.987–1.038)	0.359	0.987 (0.955–1.020)	0.428	0.998 (0.962–1.035)	0.899	0.988 (0.957–1.020)	0.457
	LAG 3	1.001 (0.976–1.027)	0.925	0.971 (0.940–1.004)	0.084	1.004 (0.968–1.041)	0.838	0.997 (0.966–1.030)	0.867
SO_2_	LAG 0	1.028 (0.980–1.079)	0.260	0.877 (0.768–1.000)	0.050	1.006 (0.967–1.047)	0.768	1.000 (0.978–1.022)	0.990
	LAG 1	1.007 (0.955–1.060)	0.805	1.106 (0.953–1.284)	0.186	0.993 (0.953–1.035)	0.744	1.002 (0.980–1.025)	0.843
	LAG 2	0.988 (0.935–1.043)	0.662	0.999 (0.863–1.156)	0.991	1.020 (0.980–1.061)	0.333	0.998 (0.975–1.021)	0.840
	LAG 3	1.028 (0.974–1.085)	0.315	0.953 (0.826–1.100)	0.512	0.974 (0.933–1.018)	0.248	1.008 (0.984–1.033)	0.511

NO_2_, nitrogen dioxide; PM_2.5_, particulate matter with a diameter of 2.5 μm or less; PM_10_ particulate matter with a diameter of 10 μm or less; SO_2_, sulfur dioxide; CO, carbon monoxide.

**Figure 2:**
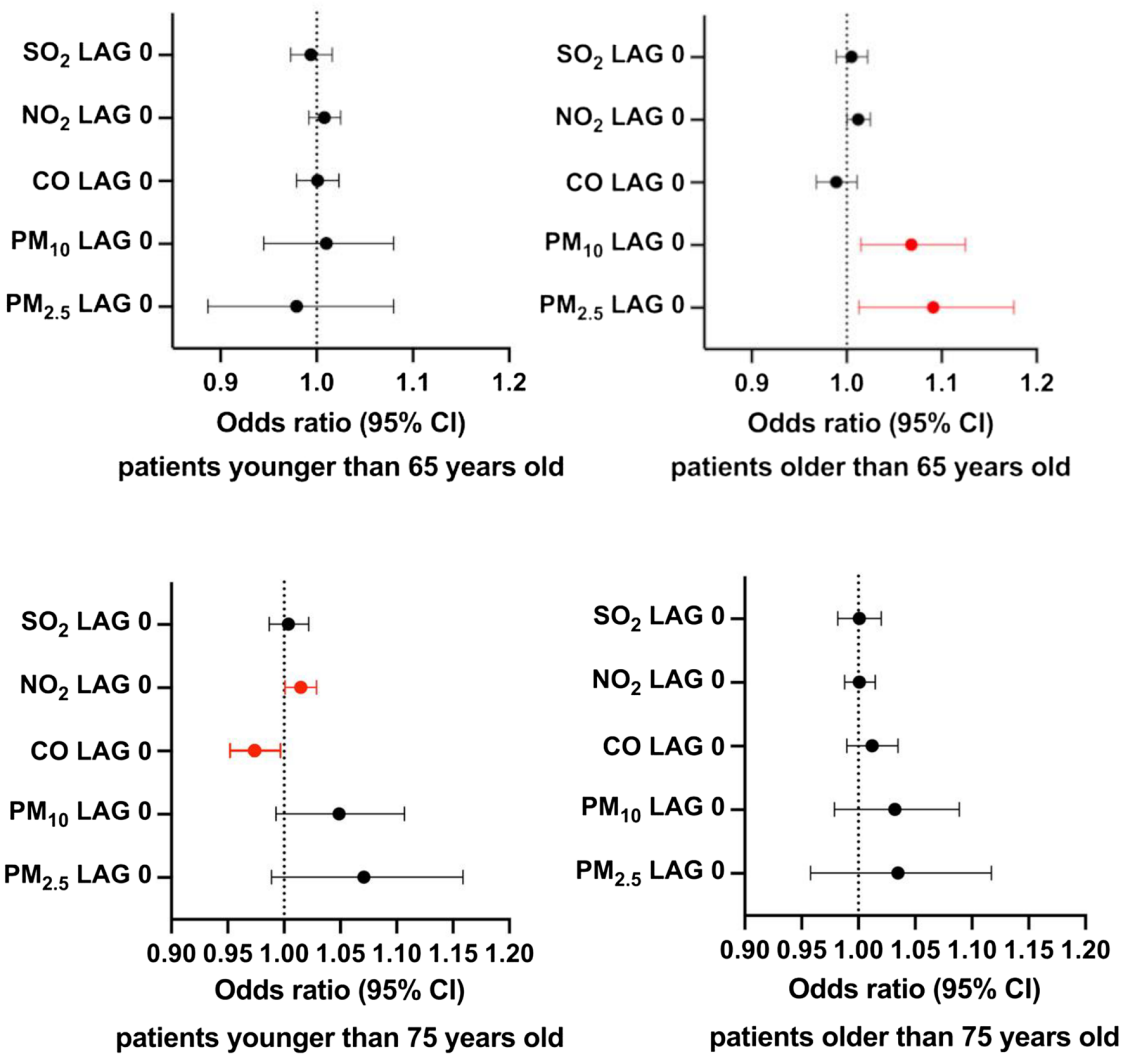
Associations between the exposure to short-term pollutants and ischemic stroke incidence in the study group divided by age.

By dividing the study group into patients above and below 75 years of age, the effect of NO_2_ and CO was observed on lag 0 (OR = 1.015, 95% CI 1.001–1.029, p = 0.038 and OR = 0.974, 95% CI 0.952–0.997, P = 0.025, respectively) in the group of patients younger than 75 years old. Moreover, when comparing people younger and older than 65 years old, there is an association between an increase in the concentration of PM_2.5_ and PM_10_ and stroke incidence in the latter group (OR = 1.091, 95% CI 1.013–1.176, P = 0.02 and OR = 1.068, 95% CI 1.015–1.125, P = 0.01, respectively). There was no significant impact of air pollution on stroke occurrence in people below 65 years old (Fig. 2).

**Figure 3:**
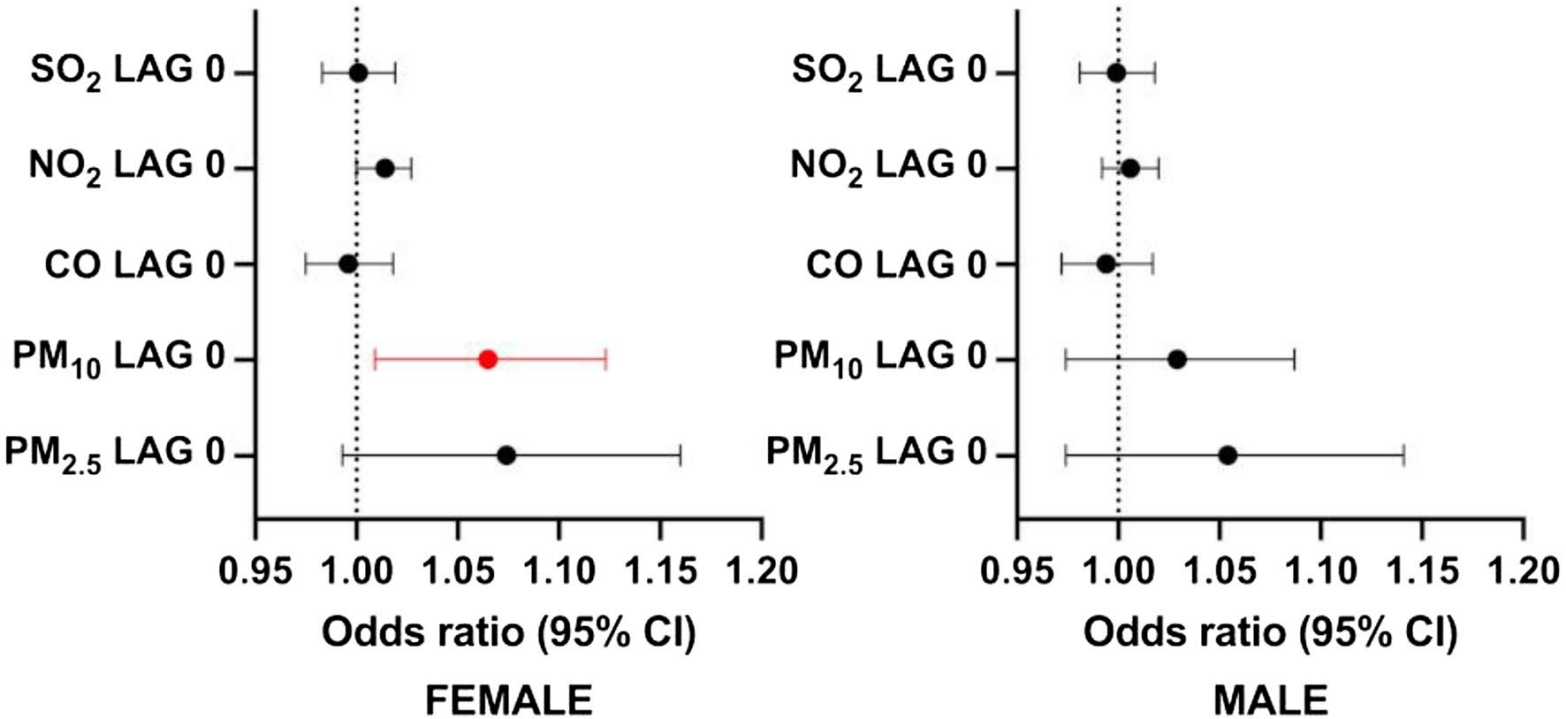
Associations between the exposure to short-term pollutants and ischemic stroke incidence in the study group divided by sex.

By dividing the study group into women and men, the effect of PM_10_ was observed on lag 0 (OR = 1.065, 95% CI 1.009–1.123, P = 0.02) in the female patients. There was no significant impact of air pollution on stroke occurrence in the male population (Fig. 3).

## Discussion

Our study is comprehensive and differs from others for several reasons. First, we analyze a non-industrial region with a predominance of the agriculture sector (considered as one of the cleanest regions in Poland). Second, we consider several types of air pollutants including PM_2.5_, PM_10_, NO_2_, SO_2,_ and CO concentrations. Third, in our analyses, we pay attention to the heterogeneity of the studied group and the analyzed period—we estimate the impact of the mentioned pollutants on stroke incidences in subgroups by age, sex, and season of the year.

The main findings of our work show that air pollution affects the incidence of ischemic strokes with the greatest effect in the winter season. The main pollutants affecting the number of hospitalizations were PMs as well as NO_2_ and CO. Considering the occurrence of strokes over a week, the highest number occurred at the beginning of the week.

Chronobiology is a trending field of science that examines how natural rhythms affect our lives. Seasonal and weekly fluctuations have a serious influence on human health, which was demonstrated multiple times^10,11,22,40^. Our study revealed significant seasonal variation in the occurrence and mortality rate of ischemic stroke. Most recent studies have shown an association between cold season and greater hospital admission and mortality due to acute ischemic stroke, which comes in line with our results^10,11^. On the other hand, Bahonar et al.^42^ and Raj et al.^43^ demonstrated no considerable seasonal fluctuation in Isfahan, Iran and New Delhi, India, respectively. However, these two aforementioned studies were performed in warm climates with relatively high temperatures during the cold season, suggesting that these conclusions may not be easily extrapolated to temperate climate countries. There are many ischemic stroke risk factors with seasonal predominance such as hypertension and atrial fibrillation. According to many studies, average blood pressure is significantly higher in the winter season due to low temperature causing vasoconstriction and increased vascular resistance^44,45^. Many analyses from different countries have shown that sympathetic nervous system stimulation and the aforementioned higher blood pressure during the cold season lead to a greater occurrence of atrial fibrillation^13^.

Our work showed that not only seasonal fluctuation matters but also weekly variation plays a huge role in ischemic stroke occurrence. In line with other publications, our results revealed that the frequency of stroke increased at the beginning of the week and decreased over the weekends^22^. To this day there is still a lack of research to explain this phenomenon. It may be related to the beginning of the week being a period with higher blood pressure and greater psychological stress compared to other days. Murakami et al.^21^ suggested morning blood pressure surge was the greatest on Mondays. On the other hand, some of the latest studies argue that incidents with similar pathomechanisms such as acute coronary syndromes might peak on other days of the week^40^. It may be due to the fact that over time more people are working from home with a much less rigid framework of a working week. In addition, as we demonstrated, short-term exposure to air pollution may be an important trigger for ischemic stroke. Reis et al.^46^ suggested that exposure to NO_2_ and PM_2.5_ varies considerably accounting for home and workplace locations with higher concentrations in the latter. Recently World Health Organization introduced new 2021 Air Quality Guidelines (2021) with more demanding thresholds^36^. The frequency of hospitalizations due to ischemic stroke on the days with exceeded daily norms of PM_2.5_ and NO_2_ was significantly higher than on days without exceeded limit values, whereas stroke occurrence was similar on the days with and without exceeded daily norms of PM_10_.

In our analysis, 54.4% of stroke events were experienced by women. That is a common pattern in Western countries due to longer life expectancy and older age at the time of stroke onset in the female population^47,48^. This can also be attributed to the loss of the protective effect of estrogen in postmenopausal elderly women^49^. However, most studies from Asia, especially China, demonstrate that men have a higher prevalence of stroke, reaching even up to 65.5% of all stroke events^50,51^. We believe it may be related to smoking and particularly disproportion between male and female smokers in Western and Asian countries. In China, 49.7% of men and only 3.5% of women smoke cigarettes, while in Poland there are 31.8% male and 24.4% female smokers^52^. Men are 14-times more likely to smoke tobacco products than women in China, whereas this difference is not that noticeable in Poland, since the smoking men-to-women ratio is only 1.3.

Our study region—Bialystok—is a non-heavily industrialized area, but a high prevalence of detached houses and coal combustion being still a popular way of household heating leads to high concentrations of PMs, especially in the cold season^53^. This may explain the seasonal results of our analysis as the effect of PMs and NO_2_ was more pronounced in the winter season. In addition, the effect of pollutants on stroke incidence has been studied in different subgroups of patients^54,55^. Zhao et al.^41^ showed that among patients with hypertension, the effects of PM_2.5_ and PM_10_ were greater during the cold season. This seasonal trend was also confirmed in an overall group of patients with stroke^56^. On the other hand, in a study performed in Tianjin, a higher risk of ischemic stroke was observed between April and September^27^.

The air pollution problem is no longer only a concern for metropolitan areas, as was once commonly believed, where we have an extensive monitoring system, and where the awareness of residents is steadily increasing. It is becoming clear that seemingly pollution-free smaller agglomerations and peripheral cities are also struggling with smog^57^. Low environmental awareness often combined with low socioeconomic status consequently leads to suboptimal ecological choices that affect the health and lives of the inhabitants of these areas. Once we are aware of this, we can notice that not enough research on the influence of air pollution on human health is performed in smaller cities, since most of the studies take place in big agglomerations^58,59^.

Even though the analyzed area is often described as the Green Lungs of Poland, the PM_2.5_ daily mean limit given by the WHO guidelines was exceeded on a considerable number of days, as we mentioned before. We found that a 10 μg/m^3^ increase in PM_10_ concentration was associated with a significant increase in stroke incidence on the day of exposure, however, no significant association for PM_2.5_ was found. Moreover, the possibility of a day without stroke was 9% for a decrease of 10 μg/m^3^ in PM_2.5_ and PM_10_ concentration. In a study on 248 Chinese cities, authors observed that a 10 μg/m^3^ increase in PM_2.5_ was significantly associated with hospital admissions due to ischemic stroke^60^. Hu et al.^56^ in a time-series study noted a 1.06% increase in stroke hospitalizations with every 10 μg/m^3^ increase in PM_2.5_. Several studies show associations between both particulate matters (PM_2.5_ and PM_10_) and ischemic stroke^61,62^. On the other hand, Dong et al.^63^ reported no significant associations between particulate matters and daily ischemic stroke counts.

In our study, we additionally wanted to determine whether there was age- and sex-dependent difference in stroke incidence. We found that females were more susceptible to exceeded PM_10_ concentrations on the day of exposure which is in line with the case-crossover analysis by Wang et al.^64^. Similar results were obtained for PM_2.5_ short-term exposure^56^. As for the age, people over the age of 65 were more vulnerable to the effects of increased concentrations of PM_2.5_ as well as PM_10_ on the day of exposure. On the contrary, Qi et al. ^27^ results suggest younger age is associated with a higher risk of pollution-triggered ischemic stroke, which is interesting considering that none of the pollutants analyzed in our study showed an impact on the group of patients under 65 years old.

Reports revealed that with 37.9%, Poland is the country with the highest share of old passenger cars (20 years or older) in the EU^6^. These older automobiles tend to have greater emissions of gaseous air pollutants such as NO_2_, SO_2,_ and CO. We observed that a 10 µg/m^3^ increase in NO_2_ concentration was associated with greater stroke incidence on the day of exposure for people under 75 years old (OR 1.015 95% CI 1.001–1.029 p = 0.038), both sexes during the winter season (OR 1.028 95% CI 1.006–1.050 p = 0.013) and also females regardless of age and season (OR 1.014 95% CI 1.000–1.027 p = 0.043). Most of the conducted analyses are coherent with each other, showing an association between short-term exposure to NO_2_ and ischemic stroke occurrence^63,64^. Moreover, Byrne et al.^61^ found that an increase in NO_2_ concentration was associated with a rise in hospitalizations due to stroke in winter. On the other hand, Sun et al.^65^ demonstrated no significant impact of NO_2_ on ischemic stroke. There is still not enough research on air pollutants’ influence on human health depending on age group, gender, and seasons. As our results have shown, we believe people in their early retirement days (younger than 75 years old) are far more mobile, spend more time outdoors, and therefore are more exposed to ambient air pollution. On the other hand, older people (older than 75 years old) tend to spend more and more time indoors each year due to various sicknesses and a lack of energy, therefore are less likely to experience exposure to air pollutants such as NO_2_. In our study, we noted that a 10 µg/m^3^ increase in CO concentration had opposite effects on LAG 2 (OR 0.976, 95% CI 0.953–0.999, p = 0.037) and LAG 3 (OR 1.026 95% CI 1.004–1.049 p = 0.022), especially LAG 2 (OR 0.957, 95% CI 0.924–0.990, p = 0.012) and LAG 3 (OR 1.037, 95% CI 1.008–1.067, p = 0.013) in summer season. We have three hypotheses regarding this occurrence. It was shown multiple times in experimental studies that carbon monoxide has a neuroprotective effect by suppressing neuroinflammation and alleviating blood–brain barrier disruption, activation of the Nrf2 pathway, improving mitochondrial biogenesis, etc.^66^. Moreover, Zeynalov et al.^67^ presented that appropriate CO levels protected the brain from transient focal ischemia after 90 min and reperfusion injury after 48 h in mice subjected to occlusion of the middle cerebral artery. Firstly, even though carbon monoxide has a harmful effect on human health, exposure to this particular air pollutant in appropriate concentrations might prove beneficial and decrease ischemic stroke incidence. Secondly, it can also be explained by a phenomenon similar to the “harvesting effect”. Shah et al.^68^ in their meta-analysis demonstrated an increase in stroke incidence on the day of exposure to CO, however, 2 days after exposure we observed a significant decrease in hospitalizations in our study. A short period of excess ischemic stroke incidence might cause a period of ischemic stroke deficit. Lastly, we assume that not only mean CO concentration have an impact on stroke incidence but also fluctuations in concentrations may have an influence on stroke occurrence. Contrary to many other studies, in our work, short-term exposure to SO_2_ had no significant impact on stroke incidence^68,69^.

Numerous studies have been conducted on the influence of air pollutants on the occurrence of ischemic stroke and there is contrasting evidence regarding the influence of individual air pollutants^68,70^. However, in our study group, several observations not previously found collectively in a single analysis emerged. Such different results of the influence of different pollutants can be partly explained by the difference in the studied regions and populations, which has a direct influence on the smog composition. The size of the study population and the period of the analysis is also not without significance.

Any study proving the harmful effects on human health should prompt the government to implement systemic changes that could significantly reduce the incidence of morbidity in the future. Promoting pro-environmental choices among residents and making them aware of the harmful effects of smog on human health and life is also significant. At this point, the fastest clinical course of action is to educate vulnerable groups and implement preventive behaviors in their lives, since the benefits of something as simple as taking a walk outside can be overshadowed by the negative effects of pollution exposure^71^.

Several strengths of our study should be noted. A large sample of patients with ischemic stroke was analyzed, additionally, the period of the analysis is noteworthy. Data on the occurrence of ischemic stroke were obtained from the main hospitals in Bialystok admitting and treating stroke patients. The analysis of a substantial number of pollutants, not only particulate matter, is also a major advantage (Supplementary Information).

## Limitations

Our study has several limitations. First, pollution data from the analyzed city came from only two existing monitoring stations. Second, since the residence was used to link with the exposure to air pollution there could be some exposure misclassification given that the address of residence may not always reflect the level of exposure of an individual.

## Conclusions

The stroke occurrence associated with air pollution was significantly greater in the winter season. The highest and the lowest frequencies of stroke incidence occurred at the beginning of the week and on weekends, respectively. Exceeded daily PM_2.5_ and NO_2_ concentration norms were associated with more admissions due to stroke. In the studied region besides PMs, the short-term influence of CO on stroke occurrence was observed. The effect was noted up to 3 days after exposure. Systemic changes are crucial and preventive measures should be undertaken for vulnerable groups, especially during wintertime.

## Supplementary Information

Supplementary Information.

## Data Availability

The data that support the findings of this study are available from the corresponding author on request.

## References

[1] World Health Organization. Global Health Estimates 2019: Deaths by Cause, Age, Sex, by Country and by Region, 2000-2019. Geneva. https://www.who.int/data/gho/data/themes/mortality-and-global-health-estimates/ghe-leading-causes-of-death (2020)

[2] GBD 2019 Stroke Collaborators (2021). Global, regional, and national burden of stroke and its risk factors, 1990–2019: A systematic analysis for the Global Burden of Disease Study 2019. Lancet Neurol..

[3] GBD 2019 Risk Factors Collaborators (2020). Global burden of 87 risk factors in 204 countries and territories, 1990–2019: A systematic analysis for the Global Burden of Disease Study 2019. The Lancet.

[4] EEA Air quality in Europe. Published 23 Nov 2020. Retrieved 14 January 2022. https://www.eea.europa.eu//publications/air-quality-in-europe-2020-report.

[5] World Bank Country and Lending Groups. Retrieved 17 May 17. https://datahelpdesk.worldbank.org/knowledgebase/articles/906519#High_income.

[6] Eurostat. Retrieved 1 January 2022. https://ec.europa.eu/eurostat/databrowser/view/road_eqs_carage/default/table?lang=en.

[7] Gangwar RS, Bevan GH, Palanivel R, Das L, Rajagopalan S (2020). Oxidative stress pathways of air pollution mediated toxicity: Recent insights. Redox Biol..

[8] Münzel T, Gori T, Al-Kindi S, Deanfield J, Lelieveld J, Daiber A, Rajagopalan S (2018). Effects of gaseous and solid constituents of air pollution on endothelial function. Eur. Heart J..

[9] Hahad O, Lelieveld J, Birklein F, Lieb K, Daiber A, Münzel T (2020). Ambient air pollution increases the risk of cerebrovascular and neuropsychiatric disorders through induction of inflammation and oxidative stress. Int. J. Mol. Sci..

[10] Fujii T, Arima H, Takashima N, Kita Y, Miyamatsu N, Tanaka-Mizuno S, Shitara S, Urushitani M, Miura K, Nozaki K (2021). Seasonal variation in incidence of stroke in a general population of 1.4 Million Japanese: The Shiga Stroke Registry. Cerebrovasc. Dis..

[11] Kurtz P, Bastos LS, Aguilar S, Hamacher S, Bozza FA (2020). Effect of seasonal and temperature variation on hospitalizations for stroke over a 10-year period in Brazil. Int. J. Stroke.

[12] Cui J, Muller MD, Blaha C, Kunselman AR, Sinoway LI (2015). Seasonal variation in muscle sympathetic nerve activity. Physiol. Rep..

[13] Loomba RS (2015). Seasonal variation in paroxysmal atrial fibrillation: A systematic review. J. Atrial Fibrill..

[14] Cichowicz R, Wielgosiński G, Fetter W (2017). Dispersion of atmospheric air pollution in summer and winter season. Environ. Monit. Assess..

[15] Shigematsu K, Watanabe Y, Nakano H (2015). Weekly variations of stroke occurrence: An observational cohort study based on the Kyoto Stroke Registry. Japan. BMJ Open.

[16] Manfredini R, Manfredini F, Fabbian F, Salmi R, Gallerani M, Bossone E, Deshmukh AJ (2016). Chronobiology of takotsubo syndrome and myocardial infarction. Heart Fail. Clin..

[17] Jakovljević D (2004). Day of the week and ischemic stroke. Stroke.

[18] Takagi H, Ando T, Mitta S, Umemoto T (2020). Meta-analysis of day-of-week variation of acute aortic rupture or dissection. J. Cardiovasc. Surg..

[19] Wallert J, Held C, Madison G, Olsson EM (2017). Temporal changes in myocardial infarction incidence rates are associated with periods of perceived psychosocial stress: A SWEDEHEART national registry study. Am. Heart J..

[20] Kimura G, Inoue N, Mizuno H, Izumi M, Nagatoya K, Ohtahara A, Munakata M (2017). Increased double product on Monday morning during work. Hypertens. Res..

[21] Murakami S, Otsuka K, Kubo Y, Shinagawa M, Yamanaka T, Ohkawa S, Kitaura Y (2004). Repeated ambulatory monitoring reveals a Monday morning surge in blood pressure in a community-dwelling population. Am. J. Hypertens..

[22] Sato T, Sakai K, Nakada R, Shiraishi T, Tanabe M, Komatsu T, Sakuta K, Terasawa Y, Umehara T, Omoto S, Mitsumura H, Murakami H, Matsushima M, Iguchi Y (2021). Employment status prior to ischemic stroke and weekly variation of stroke onset. J. Stroke Cerebrovasc. Dis..

[23] Ertl M, Beck C, Kühlbach B, Hartmann J, Hammel G, Straub A, Giemsa E, Seubert S, Philipp A, Traidl-Hoffmann C, Soentgen J, Jacobeit J, Naumann M (2019). New insights into weather and stroke: Influences of specific air masses and temperature changes on stroke incidence. Cerebrovasc. Dis..

[24] Goggins WB, Woo J, Ho S, Chan EYY, Chau PH (2011). Weather, season, and daily stroke admissions in Hong Kong. Int. J. Biometeorol..

[25] Wang X, Cao Y, Hong D, Zheng D, Richtering S, Sandset E, Leong T, Arima H, Islam S, Salam A, Anderson C, Robinson T, Hackett M (2016). Ambient temperature and stroke occurrence: A systematic review and meta-analysis. Int. J. Environ. Res. Public Health.

[26] Cao Y, Wang X, Zheng D, Robinson T, Hong D, Richtering S, Leong T, Salam A, Anderson C, Hackett M (2016). Air pressure, humidity and stroke occurrence: A systematic review and meta-analysis. Int. J. Environ. Res. Public Health.

[27] Qi X, Wang Z, Xia X, Xue J, Gu Y, Han S, Wang L, Li X, Leng SX (2020). Potential impacts of meteorological variables on acute ischemic stroke onset. Risk Manag. Healthcare Policy.

[28] Guan W, Clay SJ, Sloan GJ, Pretlow LG (2018). Effects of barometric pressure and temperature on acute ischemic stroke hospitalization in Augusta. GA. Transl. Stroke Res..

[29] World Health Organization. *WHO global air quality guidelines: Particulate matter (PM2.5 and PM10), ozone, nitrogen dioxide, sulfur dioxide and carbon monoxide*, retrieved 5 June 2022. https://apps.who.int/iris/handle/10665/345329. 34662007

[30] Local Data Bank, Statistics Poland. Retrieved on 6 June 2022. https://bdl.stat.gov.pl/bdl/dane/podgrup/tablica.

[31] Chief Inspectorate of Environmental Protection. *Annual air quality assessment in Podlaskie Voivodeship. Voivodeship report for 2018*, retrieved on June 5, 2022. https://powietrze.gios.gov.pl/pjp/documents/download/103515.

[32] Strambo D, De Marchis GM, Bonati LH, Arnold M, Carrera E, Galletta S (2022). Ischemic stroke in COVID-19 patients: Mechanisms, treatment, and outcomes in a consecutive Swiss Stroke Registry analysis. Eur. J. Neurol..

[33] Grilli, L., Rampichini, C. Ordered Logit Model. In *Encyclopedia of Quality of Life and Well-Being Research* (ed. Michalos, A.C.) (Springer, Dordrecht, 2014). 10.1007/978-94-007-0753-5_2023

[34] Wolfe R, Gould W (1998). An approximate likelihood-ratio test for ordinal response models. Stata Techn. Bull..

[35] Long JS, Freese J (2014). Regression Models for Categorical Dependent Variables Using Stata.

[36] Aidoo EN, Ackaah W (2021). A generalized ordered logit analysis of risk factors associated with driver injury severity. J. Public Health.

[37] Ugalde-Resano R, Riojas-Rodríguez H, Texcalac-Sangrador JL, Cruz JC, Hurtado-Díaz M (2022). Short term exposure to ambient air pollutants and cardiovascular emergency department visits in Mexico city. Environ. Res..

[38] Grande G, Ljungman PL, Eneroth K, Bellander T, Rizzuto D (2020). Association between cardiovascular disease and long-term exposure to air pollution with the risk of dementia. JAMA Neurol..

[39] Gasparrini A, Guo Y, Sera F, Vicedo-Cabrera AM, Huber V, Tong S, Armstrong B (2017). Projections of temperature-related excess mortality under climate change scenarios. Lancet Planet. Health.

[40] Kuźma Ł, Kurasz A, Niwińska M, Zalewska-Adamiec M, Bachórzewska-Gajewska H, Dobrzycki S (2021). Does climate change affect the chronobiological trends in the occurrence of acute coronary syndrome?. Arch. Med. Sci..

[41] Zhao Y, Guo M, An J, Zhang L, Tan P, Tian X, Luo Y (2022). Associations between ambient air pollution, meteorology, and daily hospital admissions for ischemic stroke: A time-stratified case-crossover study in Beijing. Environ. Sci. Pollut. Res..

[42] Bahonar A, Khosravi A, FariborzKhorvash F, Maracy M, Saadatnia M (2017). Seasonal and monthly variation in stroke and its subtypes-10 year hospital-based study. Mater. Soc. Med..

[43] Raj K, Bhatia R, Prasad K, Padma Srivastava MV, Vishnubhatla S, Singh MB (2015). Seasonal differences and circadian variation in stroke occurrence and stroke subtypes. J. Stroke Cerebrovasc. Dis..

[44] Narita K, Hoshide S, Kario K (2021). Seasonal variation in blood pressure: Current evidence and recommendations for hypertension management. Hypertens. Res..

[45] Kollias A, Kyriakoulis KG, Stambolliu E, Ntineri A, Anagnostopoulos I, Stergiou GS (2020). Seasonal blood pressure variation assessed by different measurement methods: Systematic review and meta-analysis. J. Hypertens..

[46] Reis S, Liška T, Vieno M, Carnell EJ, Beck R, Clemens T, Dragosits U, Tomlinson SJ, Leaver D, Heal MR (2018). The influence of residential and workday population mobility on exposure to air pollution in the UK. Environ. Int..

[47] Reeves MJ, Bushnell CD, Howard G, Gargano JW, Duncan PW, Lynch G, Khatiwoda A, Lisabeth L (2008). Sex differences in stroke: Epidemiology, clinical presentation, medical care, and outcomes. Lancet Neurol..

[48] Virani SS, Alonso A, Aparicio HJ, Benjamin EJ, Bittencourt MS, Callaway CW, Carson AP, Chamberlain AM, Cheng S, Delling FN, Elkind MSV, Evenson KR, Ferguson JF, Gupta DK, Khan SS, Kissela BM, Knutson KL, Lee CD, Lewis TT (2021). Heart disease and stroke statistics—2021 update. Circulation.

[49] Koellhoffer EC, McCullough LD (2012). The effects of estrogen in ischemic stroke. Transl. Stroke Res..

[50] Tian Y, Xiang X, Wu Y, Cao Y, Song J, Sun K, Liu H, Hu Y (2017). Fine particulate air pollution and first hospital admissions for ischemic stroke in Beijing, China. Sci. Rep..

[51] Huang F, Luo Y, Guo Y, Tao L, Xu Q, Wang C, Wang A, Li X, Guo J, Yan A, Guo X (2016). Particulate matter and hospital admissions for stroke in Beijing, China: Modification effects by ambient temperature. J. Am. Heart Assoc..

[52] GBD 2019 Tobacco Collaborators (2021). Spatial, temporal, and demographic patterns in prevalence of smoking tobacco use and attributable disease burden in 204 countries and territories, 1990–2019: A systematic analysis from the Global Burden of Disease Study 2019. The Lancet.

[53] Juda-Rezler K, Reizer M, Maciejewska K, Błaszczak B, Klejnowski K (2020). Characterization of atmospheric PM2.5 sources at a Central European urban background site. Sci. Total Env..

[54] Liu X, Li Z, Guo M, Zhang J, Tao L, Xu X, Deginet A, Lu F, Luo Y, Liu M, Liu M, Sun Y, Li H, Guo X (2021). Acute effect of particulate matter pollution on hospital admissions for stroke among patients with type 2 diabetes in Beijing, China, from 2014 to 2018. Ecotoxicol. Environ. Saf..

[55] Rhinehart ZJ, Kinnee E, Essien UR, Saul M, Guhl E, Clougherty JE, Magnani JW (2020). Association of fine particulate matter and risk of stroke in patients with atrial fibrillation. JAMA Netw. Open.

[56] Hu W, Chen Y, Chen J (2021). Short-term effect of fine particular matter on daily hospitalizations for ischemic stroke: A time-series study in Yancheng, China. Ecotoxicol. Environ. Saf..

[57] Kuźma Ł, Pogorzelski S, Struniawski K, Bachórzewska-Gajewska H, Dobrzycki S (2020). Exposure to air pollution—a trigger for myocardial infarction? A nine-year study in Bialystok—the capital of the Green Lungs of Poland (BIA-ACS registry). Int. J. Hyg. Environ. Health.

[58] Guo P, Wang Y, Feng W, Wu J, Fu C, Deng H, Huang J, Wang L, Zheng M, Liu H (2017). Ambient air pollution and risk for ischemic stroke: A short-term exposure assessment in South China. Int. J. Environ. Res. Public Health.

[59] Chen H, Cheng Z, Li M, Luo P, Duan Y, Fan J, Xu Y, Pu K, Zhou L (2022). Ambient air pollution and hospitalizations for ischemic stroke: A time series analysis using a distributed lag nonlinear model in Chongqing, China. Front. Public Health.

[60] Gu J, Shi Y, Chen N, Wang H, Chen T (2020). Ambient fine particulate matter and hospital admissions for ischemic and hemorrhagic strokes and transient ischemic attack in 248 Chinese cities. Sci. Total Env..

[61] Byrne CP, Bennett KE, Hickey A, Kavanagh P, Broderick B, O’Mahony M, Williams DJ (2020). Short-term air pollution as a risk for stroke admission: A time-series analysis. Cerebrovasc. Dis..

[62] Chen L, Zhang Y, Zhang W, Chen G, Lu P, Guo Y, Li S (2020). Short-term effect of PM1 on hospital admission for ischemic stroke: A multi-city case-crossover study in China. Environ. Pollut..

[63] Dong H, Yu Y, Yao S, Lu Y, Chen Z, Li G, Yao Y, Yao X, Wang SL, Zhang Z (2018). Acute effects of air pollution on ischaemic stroke onset and deaths: A time-series study in Changzhou, China. BMJ Open.

[64] Wang Z, Peng J, Liu P, Duan Y, Huang S, Wen Y, Liao Y, Li H, Yan S, Cheng J, Yin P (2020). Association between short-term exposure to air pollution and ischemic stroke onset: A time-stratified case-crossover analysis using a distributed lag nonlinear model in Shenzhen, China. Environ. Health Glob. Access Sci. Sourc..

[65] Sun S, Stewart JD, Eliot MN, Yanosky JD, Liao D, Tinker LF, Eaton CB, Whitsel EA, Wellenius GA (2019). Short-term exposure to air pollution and incidence of stroke in the Women’s Health Initiative. Environ. Int..

[66] Li B, Xiong J, Liu HX, Li D, Chen G (2022). Devil or angel: Two roles of carbon monoxide in stroke. Med. Gas Res..

[67] Zeynalov E, Doré S (2009). Low doses of carbon monoxide protect against experimental focal brain ischemia. Neurotox. Res..

[68] Shah AS, Lee KK, McAllister DA, Hunter A, Nair H, Whiteley W, Langrish JP, Newby DE, Mills NL (2015). Short term exposure to air pollution and stroke: Systematic review and meta-analysis. BMJ (Clin. Res. Ed.).

[69] Chen C, Wang X, Lv C, Li W, Ma D, Zhang Q, Dong L (2019). The effect of air pollution on hospitalization of individuals with respiratory and cardiovascular diseases in Jinan, China. Medicine.

[70] Verhoeven JI, Allach Y, Vaartjes I, Klijn C, de Leeuw FE (2021). Ambient air pollution and the risk of ischaemic and haemorrhagic stroke. Lancet. Planet. Health.

[71] Yang Z, Wu M, Lu J, Gao K, Yu Z, Li T, Liu W, Shen P, Lin H, Shui L, Tang M, Jin M, Chen K, Wang J (2022). Interaction between walkability and fine particulate matter on risk of ischemic stroke: A prospective cohort study in China. Env. Pollut. (Barking Essex: 1987).

