# Supplementary material for: Exposure to air pollution and its effect on ischemic strokes (EP-PARTICLES study)
Source: Sci Rep. 2022 Oct 13;12:17150. doi: 10.1038/s41598-022-21585-7 (PMC9563068; doi:10.1038/s41598-022-21585-7)
Supplement: Supplementary file 1 — Supplementary Information. [file 41598_2022_21585_MOESM1_ESM.docx]

| **Variables** | **PM2.5** | **PM10** | **NO2** | **SO2** | **CO** |
| --- | --- | --- | --- | --- | --- |
| **LAG 0** | | | | | |
| OR | 1.075 | 1.056 | 1.013 | 0.999 | 0.989 |
| 95% CI | 0.999-1.157 | 1.004-1.110 | 1.000-1.025 | 0.983-1.016 | 0.969-1.011 |
| p-value | 0.053 | 0.035 | 0.052 | 0.925 | 0.325 |
| **LAG 1** | | | | | |
| OR | 0.975 | 0.981 | 0.993 | 1.002 | 1.016 |
| 95% CI | 0.894-1.062 | 0.923-1.042 | 0.979-1.008 | 0.985-1.020 | 0.991-1.042 |
| p-value | 0.557 | 0.533 | 0.371 | 0.828 | 0.213 |
| **LAG 2** | | | | | |
| OR | 0.984 | 0.963 | 0.996 | 0.999 | 0.976 |
| 95% CI | 0.902-1.074 | 0.907-1.023 | 0.982-1.011 | 0.981-1.016 | 0.953-0.999 |
| p-value | 0.718 | 0.224 | 0.624 | 0.873 | 0.037 |
| **LAG 3** | | | | | |
| OR | 1.015 | 1.024 | 0.999 | 1.002 | 1.026 |
| 95% CI | 0.929-1.110 | 0.962-1.089 | 0.984-1.014 | 0.984-1.020 | 1.004-1.049 |
| p-value | 0.739 | 0.454 | 0.878 | 0.818 | 0.022 |
| **LAG 4** | | | | | |
| OR | 1.021 | 0.996 | 1.002 | 1.01 | 0.996 |
| 95% CI | 0.934-1.115 | 0.937-1.060 | 0.987-1.017 | 0.993-1.027 | 0.975-1.017 |
| p-value | 0.652 | 0.908 | 0.797 | 0.266 | 0.677 |
| **LAG 5** | | | | | |
| OR | 0.995 | 1.015 | 1.004 | 0.994 | 1.011 |
| 95% CI | 0.912-1.085 | 0.956-1.077 | 0.989-1.019 | 0.977-1.010 | 0.990-1.033 |
| p-value | 0.905 | 0.636 | 0.593 | 0.453 | 0.31 |
| **LAG 6** | | | | | |
| OR | 0.989 | 1.002 | 0.998 | 1 | 0.987 |
| 95% CI | 0.908-1.076 | 0.944-1.064 | 0.984-1.013 | 0.981-1.019 | 0.965-1.009 |
| p-value | 0.789 | 0.944 | 0.801 | 0.997 | 0.24 |
| **LAG 7** | | | | | |
| OR | 1.012 | 0.996 | 1.007 | 1.007 | 1.007 |
| 95% CI | 0.943-1.085 | 0.948-1.046 | 0.994-1.020 | 0.991-1.023 | 0.987-1.026 |
| p-value | 0.749 | 0.859 | 0.278 | 0.409 | 0.504 |

**Supplementary Appendix**

Table A1. Overall associations between the exposure to short-term pollutants and ischemic stroke incidence.

Abbreviations: NO_2_, nitrogen dioxide; PM_2.5_, particulate matter with a diameter of 2.5 μm or less; PM_10_ particulate matter with a diameter of 10 μm or less; SO_2_, sulfur dioxide; CO, carbon monoxide; OR, odds ratio; 95% CI, 95% Confidence Intervals.

Table A2. Associations between the exposure to short-term pollutants and ischemic stroke incidence in female population.

| **Variables** | **PM2.5** | **PM10** | **NO2** | **SO2** | **CO** |
| --- | --- | --- | --- | --- | --- |
| **LAG 0** | | | | | |
| OR | 1.074 | 1.065 | 1.014 | 1.001 | 0.996 |
| 95% CI | 0.993-1.160 | 1.009-1.123 | 1.000-1.027 | 0.983-1.019 | 0.975-1.018 |
| p-value | 0.073 | 0.022 | 0.043 | 0.885 | 0.724 |
| **LAG 1** | | | | | |
| OR | 0.951 | 0.959 | 0.996 | 1.013 | 1.001 |
| 95% CI | 0.867-1.042 | 0.900-1.023 | 0.981-1.012 | 0.995-1.032 | 0.977-1.025 |
| p-value | 0.279 | 0.204 | 0.631 | 0.153 | 0.932 |
| **LAG 2** | | | | | |
| OR | 1.027 | 0.993 | 1.001 | 1 | 0.985 |
| 95% CI | 0.936-1.126 | 0.931-1.060 | 0.986-1.017 | 0.981-1.019 | 0.962-1.010 |
| p-value | 0.577 | 0.84 | 0.862 | 0.985 | 0.241 |
| **LAG 3** | | | | | |
| OR | 0.993 | 1.004 | 0.992 | 0.992 | 1.020 |
| 95% CI | 0.904-1.090 | 0.941-1.071 | 0.977-1.007 | 0.972-1.012 | 0.997-1.044 |
| p-value | 0.877 | 0.908 | 0.309 | 0.41 | 0.084 |
| **LAG 4** | | | | |  |
| OR | 1.005 | 0.982 | 0.998 | 1.012 | 1.01 |
| 95% CI | 0.917-1.103 | 0.920-1.047 | 0.983-1.013 | 0.993-1.032 | 0.987-1.032 |
| p-value | 0.911 | 0.576 | 0.76 | 0.215 | 0.397 |
| **LAG 5** | | | | | |
| OR | 0.941 | 0.994 | 0.998 | 0.996 | 0.985 |
| 95% CI | 0.858-1.031 | 0.932-1.059 | 0.983-1.013 | 0.978-1.014 | 0.962-1.009 |
| p-value | 0.19 | 0.84 | 0.776 | 0.638 | 0.217 |
| **LAG 6** | | | | | |
| OR | 1.037 | 1.027 | 1.005 | 0.994 | 1.001 |
| 95% CI | 0.947-1.135 | 0.964-1.093 | 0.990-1.021 | 0.976-1.013 | 0.978-1.025 |
| p-value | 0.431 | 0.415 | 0.506 | 0.553 | 0.941 |
| **LAG 7** | | | | | |
| OR | 0.984 | 0.983 | 1.005 | 1.002 | 1.004 |
| 95% CI | 0.913-1.060 | 0.932-1.037 | 0.992-1.019 | 0.986-1.020 | 0.984-1.025 |
| p-value | 0.671 | 0.531 | 0.431 | 0.776 | 0.678 |

Abbreviations: NO_2_, nitrogen dioxide; PM_2.5_, particulate matter with a diameter of 2.5 μm or less; PM_10_ particulate matter with a diameter of 10 μm or less; SO_2_, sulfur dioxide; CO, carbon monoxide; OR, odds ratio; 95% CI, 95% Confidence Intervals.

Table A3. Associations between the exposure to short-term pollutants and ischemic stroke incidence in male population.

| **Variables** | **PM2.5** | **PM10** | **NO2** | **SO2** | **CO** |
| --- | --- | --- | --- | --- | --- |
| **LAG 0** |  |  |  |  |  |
| OR | 1.054 | 1.029 | 1.006 | 0.999 | 0.994 |
| 95% CI | 0.974-1.141 | 0.974-1.087 | 0.992-1.020 | 0.981-1.018 | 0.972-1.017 |
| p-value | 0.19 | 0.308 | 0.391 | 0.924 | 0.599 |
| **LAG 1** | | | | | |
| OR | 0.987 | 1.007 | 0.99 | 0.988 | 1.016 |
| 95% CI | 0.900-1.082 | 0.944-1.074 | 0.975-1.006 | 0.967-1.010 | 0.989-1.045 |
| p-value | 0.78 | 0.827 | 0.205 | 0.284 | 0.253 |
| **LAG 2** | | | | | |
| OR | 0.977 | 0.961 | 0.995 | 1.003 | 0.974 |
| 95% CI | 0.890-1.072 | 0.900-1.027 | 0.979-1.011 | 0.983-1.023 | 0.948-1.000 |
| p-value | 0.621 | 0.241 | 0.521 | 0.773 | 0.05 |
| **LAG 3** | | | | | |
| OR | 0.993 | 1.003 | 1.003 | 1.003 | 1.024 |
| 95% CI | 0.902-1.093 | 0.938-1.073 | 0.988-1.020 | 0.981-1.025 | 0.998-1.050 |
| p-value | 0.882 | 0.929 | 0.677 | 0.802 | 0.07 |
| **LAG 4** | | | | | |
| OR | 1.004 | 1.012 | 1.004 | 1.006 | 0.982 |
| 95% CI | 0.913-1.105 | 0.948-1.082 | 0.989-1.020 | 0.986-1.027 | 0.956-1.008 |
| p-value | 0.933 | 0.714 | 0.592 | 0.539 | 0.181 |
| **LAG 5** | | | | | |
| OR | 1.037 | 1.021 | 1.007 | 0.993 | 1.031 |
| 95% CI | 0.944-1.139 | 0.956-1.091 | 0.991-1.023 | 0.974-1.013 | 1.005-1.058 |
| p-value | 0.451 | 0.531 | 0.382 | 0.501 | 0.019 |
| **LAG 6** | | | | | |
| OR | 0.963 | 0.99 | 0.99 | 1.004 | 0.982 |
| 95% CI | 0.879-1.055 | 0.929-1.056 | 0.975-1.006 | 0.985-1.024 | 0.958-1.006 |
| p-value | 0.415 | 0.763 | 0.228 | 0.66 | 0.134 |
| **LAG 7** | | | | | |
| OR | 1.032 | 1.012 | 1.008 | 1.009 | 1.006 |
| 95% CI | 0.957-1.114 | 0.959-1.067 | 0.994-1.022 | 0.992-1.026 | 0.985-1.027 |
| p-value | 0.411 | 0.666 | 0.275 | 0.321 | 0.581 |

Abbreviations: NO_2_, nitrogen dioxide; PM_2.5_, particulate matter with a diameter of 2.5 μm or less; PM_10_ particulate matter with a diameter of 10 μm or less; SO_2_, sulfur dioxide; CO, carbon monoxide; OR, odds ratio; 95% CI, 95% Confidence Intervals.

Table A4. Associations between the exposure to short-term pollutants and ischemic stroke incidence in the study group of people younger than 65 years old.

| **Variables** | **PM2.5** | **PM10** | **NO2** | **SO2** | **CO** |
| --- | --- | --- | --- | --- | --- |
| **LAG 0** | | | | | |
| OR | 0.979 | 1.01 | 1.008 | 0.994 | 1.001 |
| 95% CI | 0.887-1.080 | 0.945-1.080 | 0.992-1.025 | 0.973-1.016 | 0.979-1.023 |
| p-value | 0.667 | 0.767 | 0.343 | 0.589 | 0.934 |
| **LAG 1** | | | | | |
| OR | 0.984 | 0.96 | 0.985 | 1.01 | 1.022 |
| 95% CI | 0.878-1.102 | 0.886-1.041 | 0.966-1.004 | 0.989-1.032 | 0.994-1.051 |
| p-value | 0.778 | 0.325 | 0.113 | 0.339 | 0.126 |
| **LAG 2** | | | | | |
| OR | 1.011 | 1.037 | 1 | 0.979 | 0.974 |
| 95% CI | 0.904-1.132 | 0.959-1.122 | 0.981-1.019 | 0.955-1.004 | 0.944-1.006 |
| p-value | 0.844 | 0.357 | 0.994 | 0.1 | 0.106 |
| **LAG 3** | | | | | |
| OR | 0.963 | 0.958 | 1.001 | 1.020 | 1.004 |
| 95% CI | 0.858-1.081 | 0.884-1.039 | 0.982-1.021 | 0.998-1.042 | 0.975-1.033 |
| p-value | 0.523 | 0.304 | 0.899 | 0.076 | 0.806 |
| **LAG 4** | | | | | |
| OR | 0.937 | 0.975 | 0.992 | 0.992 | 0.984 |
| 95% CI | 0.835-1.052 | 0.900-1.055 | 0.974-1.012 | 0.969-1.017 | 0.953-1.015 |
| p-value | 0.273 | 0.527 | 0.433 | 0.538 | 0.3 |
| **LAG 5** | | | | | |
| OR | 1.035 | 1.034 | 1.002 | 1.009 | 1.028 |
| 95% CI | 0.927-1.156 | 0.958-1.116 | 0.983-1.021 | 0.988-1.030 | 0.999-1.058 |
| p-value | 0.538 | 0.395 | 0.844 | 0.391 | 0.057 |
| **LAG 6** | | | | | |
| OR | 0.994 | 0.992 | 0.991 | 0.995 | 0.986 |
| 95% CI | 0.891-1.108 | 0.918-1.071 | 0.972-1.009 | 0.974-1.016 | 0.958-1.015 |
| p-value | 0.908 | 0.83 | 0.329 | 0.614 | 0.351 |
| **LAG 7** | | | | | |
| OR | 1.047 | 1.025 | 1.014 | 1.022 | 1.007 |
| 95% CI | 0.957-1.145 | 0.962-1.092 | 0.997-1.030 | 1.004-1.041 | 0.982-1.033 |
| p-value | 0.318 | 0.442 | 0.107 | 0.015 | 0.572 |

Abbreviations: NO_2_, nitrogen dioxide; PM_2.5_, particulate matter with a diameter of 2.5 μm or less; PM_10_ particulate matter with a diameter of 10 μm or less; SO_2_, sulfur dioxide; CO, carbon monoxide; OR, odds ratio; 95% CI, 95% Confidence Intervals.

Table A5. Associations between the exposure to short-term pollutants and ischemic stroke incidence in the study group of people older than 65 years old.

| **Variables** | **PM2.5** | **PM10** | **NO2** | **SO2** | **CO** |
| --- | --- | --- | --- | --- | --- |
| **LAG 0** | | | | | |
| OR | 1.091 | 1.068 | 1.012 | 1.005 | 0.989 |
| 95% CI | 1.013-1.176 | 1.015-1.125 | 1.000-1.025 | 0.989-1.022 | 0.968-1.011 |
| p-value | 0.021 | 0.012 | 0.059 | 0.529 | 0.339 |
| **LAG 1** | | | | | |
| OR | 0.965 | 0.978 | 0.995 | 0.998 | 1.004 |
| 95% CI | 0.884-1.053 | 0.920-1.040 | 0.981-1.010 | 0.979-1.017 | 0.980-1.030 |
| p-value | 0.418 | 0.484 | 0.526 | 0.809 | 0.726 |
| **LAG 2** | | | | | |
| OR | 0.982 | 0.958 | 0.997 | 1.006 | 0.984 |
| 95% CI | 0.898-1.074 | 0.901-1.020 | 0.982-1.012 | 0.988-1.024 | 0.960-1.007 |
| p-value | 0.691 | 0.179 | 0.675 | 0.52 | 0.176 |
| **LAG 3** | | | | | |
| OR | 1.024 | 1.031 | 0.999 | 0.994 | 1.027 |
| 95% CI | 0.935-1.121 | 0.968-1.099 | 0.984-1.014 | 0.976-1.012 | 1.004-1.051 |
| p-value | 0.612 | 0.337 | 0.893 | 0.477 | 0.02 |
| **LAG 4** | | | | | |
| OR | 1.042 | 1.005 | 1.005 | 1.016 | 0.999 |
| 95% CI | 0.953-1.140 | 0.944-1.070 | 0.990-1.020 | 0.998-1.034 | 0.978-1.021 |
| p-value | 0.366 | 0.876 | 0.528 | 0.082 | 0.952 |
| **LAG 5** | | | | | |
| OR | 0.959 | 0.996 | 1.002 | 0.986 | 1.006 |
| 95% CI | 0.879-1.046 | 0.937-1.059 | 0.987-1.017 | 0.968-1.004 | 0.985-1.028 |
| p-value | 0.347 | 0.907 | 0.814 | 0.131 | 0.588 |
| **LAG 6** | | | | | |
| OR | 1.006 | 1.016 | 1.001 | 1.006 | 0.994 |
| 95% CI | 0.924-1.096 | 0.956-1.079 | 0.986-1.015 | 0.987-1.025 | 0.973-1.016 |
| p-value | 0.888 | 0.614 | 0.916 | 0.562 | 0.612 |
| **LAG 7** | | | | | |
| OR | 0.993 | 0.988 | 1.003 | 0.994 | 1.001 |
| 95% CI | 0.925-1.065 | 0.940-1.039 | 0.991-1.017 | 0.978-1.011 | 0.982-1.021 |
| p-value | 0.835 | 0.649 | 0.6 | 0.496 | 0.893 |

Abbreviations: NO_2_, nitrogen dioxide; PM_2.5_, particulate matter with a diameter of 2.5 μm or less; PM_10_ particulate matter with a diameter of 10 μm or less; SO_2_, sulfur dioxide; CO, carbon monoxide; OR, odds ratio; 95% CI, 95% Confidence Intervals.

Table A6. Associations between the exposure to short-term pollutants and ischemic stroke incidence in the study group of people younger than 75 years old.

| **Variables** | **PM2.5** | **PM10** | **NO2** | **SO2** | **CO** |
| --- | --- | --- | --- | --- | --- |
| **LAG 0** | | | | | |
| OR | 1.071 | 1.049 | 1.015 | 1.004 | 0.974 |
| 95% CI | 0.989-1.159 | 0.993-1.107 | 1.001-1.029 | 0.987-1.022 | 0.952-0.997 |
| p-value | 0.09 | 0.085 | 0.038 | 0.621 | 0.025 |
| **LAG 1** | | | | | |
| OR | 0.999 | 0.998 | 0.994 | 1.006 | 1.034 |
| 95% CI | 0.910-1.096 | 0.935-1.066 | 0.979-1.010 | 0.987-1.024 | 1.008-1.061 |
| p-value | 0.978 | 0.959 | 0.49 | 0.546 | 0.009 |
| **LAG 2** | | | | | |
| OR | 0.977 | 0.993 | 0.998 | 0.991 | 0.981 |
| 95% CI | 0.890-1.074 | 0.930-1.061 | 0.982-1.014 | 0.971-1.011 | 0.956-1.006 |
| p-value | 0.634 | 0.832 | 0.782 | 0.385 | 0.139 |
| **LAG 3** | | | | | |
| OR | 1.027 | 1.009 | 1.002 | 1.007 | 1.004 |
| 95% CI | 0.935-1.128 | 0.945-1.077 | 0.986-1.018 | 0.986-1.028 | 0.980-1.028 |
| p-value | 0.577 | 0.793 | 0.836 | 0.497 | 0.772 |
| **LAG 4** | | | | | |
| OR | 1.003 | 1.017 | 1.002 | 1.001 | 1.011 |
| 95% CI | 0.913-1.101 | 0.952-1.086 | 0.986-1.018 | 0.982-1.021 | 0.985-1.037 |
| p-value | 0.955 | 0.62 | 0.817 | 0.889 | 0.411 |
| **LAG 5** | | | | | |
| OR | 1.042 | 1.022 | 1.003 | 1.002 | 1.017 |
| 95% CI | 0.949-1.145 | 0.957-1.090 | 0.988-1.019 | 0.983-1.021 | 0.991-1.042 |
| p-value | 0.387 | 0.523 | 0.677 | 0.851 | 0.198 |
| **LAG 6** | | | | | |
| OR | 0.938 | 0.973 | 0.993 | 0.992 | 0.979 |
| 95% CI | 0.854-1.030 | 0.911-1.039 | 0.977-1.009 | 0.972-1.012 | 0.956-1.003 |
| p-value | 0.178 | 0.407 | 0.379 | 0.443 | 0.093 |
| **LAG 7** | | | | | |
| OR | 1.041 | 1.014 | 1.014 | 1.013 | 1.009 |
| 95% CI | 0.964-1.124 | 0.961-1.070 | 1.000-1.028 | 0.996-1.031 | 0.988-1.029 |
| p-value | 0.304 | 0.601 | 0.048 | 0.124 | 0.417 |

Abbreviations: NO_2_, nitrogen dioxide; PM_2.5_, particulate matter with a diameter of 2.5 μm or less; PM_10_ particulate matter with a diameter of 10 μm or less; SO_2_, sulfur dioxide; CO, carbon monoxide; OR, odds ratio; 95% CI, 95% Confidence Intervals.

Table A7. Associations between the exposure to short-term pollutants and ischemic stroke incidence in the study group of people older than 75 years old.

| **Variables** | **PM2.5** | **PM10** | **NO2** | **SO2** | **CO** |
| --- | --- | --- | --- | --- | --- |
| **LAG 0** | | | | | |
| OR | 1.035 | 1.032 | 1.001 | 1.001 | 1.012 |
| 95% CI | 0.958-1.117 | 0.979-1.089 | 0.988-1.015 | 0.982-1.020 | 0.990-1.035 |
| p-value | 0.383 | 0.237 | 0.838 | 0.929 | 0.286 |
| **LAG 1** | | | | | |
| OR | 0.965 | 0.968 | 0.994 | 0.999 | 0.985 |
| 95% CI | 0.882-1.056 | 0.908-1.031 | 0.979-1.010 | 0.979-1.019 | 0.959-1.011 |
| p-value | 0.437 | 0.311 | 0.466 | 0.889 | 0.252 |
| **LAG 2** | | | | | |
| OR | 0.988 | 0.959 | 0.997 | 1.005 | 0.978 |
| 95% CI | 0.903-1.082 | 0.900-1.022 | 0.982-1.012 | 0.986-1.024 | 0.953-1.004 |
| p-value | 0.799 | 0.196 | 0.7 | 0.615 | 0.093 |
| **LAG 3** | | | | | |
| OR | 1.005 | 1.023 | 1 | 0.991 | 1.039 |
| 95% CI | 0.916-1.103 | 0.959-1.092 | 0.985-1.015 | 0.970-1.012 | 1.014-1.065 |
| p-value | 0.908 | 0.489 | 0.998 | 0.375 | 0.002 |
| **LAG 4** | | | | | |
| OR | 1.029 | 0.985 | 1.001 | 1.017 | 0.987 |
| 95% CI | 0.939-1.127 | 0.924-1.051 | 0.986-1.017 | 0.996-1.037 | 0.963-1.012 |
| p-value | 0.547 | 0.649 | 0.861 | 0.107 | 0.303 |
| **LAG 5** | | | | | |
| OR | 0.941 | 0.999 | 1.001 | 0.988 | 1.002 |
| 95% CI | 0.860-1.030 | 0.937-1.064 | 0.986-1.016 | 0.968-1.008 | 0.978-1.026 |
| p-value | 0.185 | 0.964 | 0.911 | 0.223 | 0.884 |
| **LAG 6** | | | | | |
| OR | 1.043 | 1.029 | 1.002 | 1.005 | 1.003 |
| 95% CI | 0.955-1.140 | 0.965-1.096 | 0.986-1.017 | 0.986-1.024 | 0.979-1.027 |
| p-value | 0.347 | 0.383 | 0.844 | 0.598 | 0.828 |
| **LAG 7** | | | | | |
| OR | 0.982 | 0.982 | 0.997 | 0.994 | 0.997 |
| 95% CI | 0.913-1.056 | 0.932-1.034 | 0.983-1.010 | 0.976-1.012 | 0.977-1.018 |
| p-value | 0.628 | 0.488 | 0.642 | 0.536 | 0.795 |

Abbreviations: NO_2_, nitrogen dioxide; PM_2.5_, particulate matter with a diameter of 2.5 μm or less; PM_10_ particulate matter with a diameter of 10 μm or less; SO_2_, sulfur dioxide; CO, carbon monoxide; OR, odds ratio; 95% CI, 95% Confidence Intervals.

Table A8. Associations between the exposure to short-term pollutants and ischemic stroke incidence in the spring

| **Variables** | **PM2.5** | **PM10** | **NO2** | **SO2** | **CO** |
| --- | --- | --- | --- | --- | --- |
| **LAG 0** | | | | | |
| OR | 1.091 | 1.094 | 0.998 | 0.877 | 0.908 |
| 95% CI | 0.775-1.537 | 0.880-1.360 | 0.967-1.030 | 0.768-1.000 | 0.078-10.632 |
| p-value | 0.617 | 0.42 | 0.897 | 0.05 | 0.939 |
| **LAG 1** | | | | | |
| OR | 1.332 | 1.056 | 1.008 | 1.106 | 2.51 |
| 95% CI | 0.934-1.900 | 0.843-1.322 | 0.976-1.042 | 0.953-1.284 | 0.150-42.033 |
| p-value | 0.113 | 0.635 | 0.619 | 0.186 | 0.522 |
| **LAG 2** | | | | | |
| OR | 0.652 | 0.818 | 0.987 | 0.999 | 0.141 |
| 95% CI | 0.456-0.933 | 0.655-1.022 | 0.955-1.020 | 0.863-1.156 | 0.008-2.410 |
| p-value | 0.019 | 0.077 | 0.428 | 0.991 | 0.176 |
| **LAG 3** | | | | | |
| OR | 1.125 | 0.995 | 0.971 | 0.953 | 0.319 |
| 95% CI | 0.821-1.541 | 0.804-1.233 | 0.940-1.004 | 0.826-1.100 | 0.021-4.830 |
| p-value | 0.464 | 0.966 | 0.084 | 0.512 | 0.41 |
| **LAG 4** | | | | | |
| OR | 0.847 | 0.934 | 1 | 1.037 | 1.129 |
| 95% CI | 0.621-1.155 | 0.757-1.152 | 0.969-1.033 | 0.899-1.197 | 0.072-17.742 |
| p-value | 0.294 | 0.523 | 0.976 | 0.614 | 0.931 |
| **LAG 5** | | | | | |
| OR | 0.905 | 0.869 | 1 | 0.926 | 1.212 |
| 95% CI | 0.660-1.242 | 0.702-1.076 | 0.968-1.034 | 0.800-1.073 | 0.075-19.591 |
| p-value | 0.536 | 0.198 | 0.985 | 0.306 | 0.892 |
| **LAG 6** | | | | | |
| OR | 1.305 | 1.18 | 0.994 | 1.137 | 1.829 |
| 95% CI | 0.963-1.769 | 0.962-1.446 | 0.962-1.027 | 0.984-1.314 | 0.144-23.226 |
| p-value | 0.086 | 0.111 | 0.713 | 0.081 | 0.642 |
| **LAG 7** | | | | | |
| OR | 0.927 | 1.025 | 1.023 | 1.005 | 1.972 |
| 95% CI | 0.723-1.188 | 0.872-1.205 | 0.994-1.053 | 0.888-1.137 | 0.236-16.516 |
| p-value | 0.548 | 0.767 | 0.122 | 0.943 | 0.531 |

Abbreviations: NO_2_, nitrogen dioxide; PM_2.5_, particulate matter with a diameter of 2.5 μm or less; PM_10_ particulate matter with a diameter of 10 μm or less; SO_2_, sulfur dioxide; CO, carbon monoxide; OR, odds ratio; 95% CI, 95% Confidence Intervals.

Table A9. Associations between the exposure to short-term pollutants and ischemic stroke incidence in the summer.

| **Variables** | **PM2.5** | **PM10** | **NO2** | **SO2** | **CO** |
| --- | --- | --- | --- | --- | --- |
| **LAG 0** | | | | | |
| OR | 1.218 | 1.02 | 1.016 | 1.006 | 0.995 |
| 95% CI | 0.789-1.881 | 0.801-1.298 | 0.984-1.049 | 0.967-1.047 | 0.965-1.026 |
| p-value | 0.373 | 0.873 | 0.33 | 0.768 | 0.741 |
| **LAG 1** | | | | | |
| OR | 0.631 | 1.055 | 0.987 | 0.993 | 1.019 |
| 95% CI | 0.389-1.024 | 0.799-1.392 | 0.951-1.025 | 0.953-1.035 | 0.981-1.059 |
| p-value | 0.062 | 0.706 | 0.494 | 0.744 | 0.331 |
| **LAG 2** | | | | | |
| OR | 0.863 | 0.839 | 0.998 | 1.02 | 0.957 |
| 95% CI | 0.527-1.413 | 0.635-1.110 | 0.962-1.035 | 0.980-1.061 | 0.924-0.990 |
| p-value | 0.558 | 0.219 | 0.899 | 0.333 | 0.012 |
| **LAG 3** | | | | | |
| OR | 0.785 | 1.006 | 1.004 | 0.974 | 1.037 |
| 95% CI | 0.476-1.295 | 0.750-1.348 | 0.968-1.041 | 0.933-1.018 | 1.008-1.067 |
| p-value | 0.343 | 0.971 | 0.838 | 0.248 | 0.013 |
| **LAG 4** | | | | | |
| OR | 1.129 | 0.923 | 0.982 | 1.008 | 1 |
| 95% CI | 0.678-1.878 | 0.692-1.231 | 0.947-1.019 | 0.965-1.052 | 0.973-1.027 |
| p-value | 0.641 | 0.584 | 0.346 | 0.717 | 0.979 |
| **LAG 5** | | | | | |
| OR | 0.795 | 0.936 | 0.996 | 0.993 | 1.007 |
| 95% CI | 0.473-1.336 | 0.695-1.262 | 0.960-1.035 | 0.959-1.029 | 0.981-1.034 |
| p-value | 0.386 | 0.666 | 0.852 | 0.709 | 0.596 |
| **LAG 6** | | | | | |
| OR | 1.337 | 0.981 | 1.004 | 1.028 | 0.999 |
| 95% CI | 0.790-2.263 | 0.726-1.326 | 0.968-1.043 | 0.994-1.063 | 0.970-1.030 |
| p-value | 0.279 | 0.899 | 0.817 | 0.106 | 0.972 |
| **LAG 7** | | | | | |
| OR | 1.022 | 1.135 | 1.011 | 1 | 0.998 |
| 95% CI | 0.645-1.618 | 0.873-1.474 | 0.977-1.045 | 0.968-1.033 | 0.972-1.026 |
| p-value | 0.927 | 0.344 | 0.542 | 0.998 | 0.905 |

Abbreviations: NO_2_, nitrogen dioxide; PM_2.5_, particulate matter with a diameter of 2.5 μm or less; PM_10_ particulate matter with a diameter of 10 μm or less; SO_2_, sulfur dioxide; CO, carbon monoxide; OR, odds ratio; 95% CI, 95% Confidence Intervals.

Table A10. Associations between the exposure to short-term pollutants and ischemic stroke incidence in the autumn.

| **Variables** | **PM2.5** | **PM10** | **NO2** | **SO2** | **CO** |
| --- | --- | --- | --- | --- | --- |
| **LAG 0** | | | | | |
| OR | 1.055 | 1.041 | 1.007 | 1 | 0.970 |
| 95% CI | 0.913-1.219 | 0.951-1.139 | 0.980-1.036 | 0.978-1.022 | 0.935-1.005 |
| p-value | 0.467 | 0.381 | 0.611 | 0.99 | 0.096 |
| **LAG 1** | | | | | |
| OR | 1.006 | 1.029 | 1.004 | 1.002 | 1.015 |
| 95% CI | 0.852-1.189 | 0.927-1.141 | 0.973-1.037 | 0.980-1.025 | 0.976-1.057 |
| p-value | 0.941 | 0.595 | 0.786 | 0.843 | 0.448 |
| **LAG 2** | | | | | |
| OR | 1.008 | 0.911 | 0.988 | 0.998 | 0.997 |
| 95% CI | 0.848-1.199 | 0.818-1.014 | 0.957-1.020 | 0.975-1.021 | 0.959-1.037 |
| p-value | 0.924 | 0.089 | 0.457 | 0.84 | 0.885 |
| **LAG 3** | | | | | |
| OR | 0.964 | 1.038 | 0.997 | 1.008 | 1.019 |
| 95% CI | 0.812-1.145 | 0.932-1.156 | 0.966-1.030 | 0.984-1.033 | 0.979-1.061 |
| p-value | 0.677 | 0.492 | 0.867 | 0.511 | 0.351 |
| **LAG 4** | | | | | |
| OR | 0.943 | 0.959 | 0.989 | 1.008 | 0.972 |
| 95% CI | 0.796-1.115 | 0.863-1.066 | 0.958-1.020 | 0.985-1.031 | 0.934-1.011 |
| p-value | 0.491 | 0.438 | 0.477 | 0.506 | 0.155 |
| **LAG 5** | | | | | |
| OR | 1.094 | 1.066 | 1.02 | 0.988 | 1.022 |
| 95% CI | 0.925-1.294 | 0.960-1.184 | 0.989-1.053 | 0.966-1.011 | 0.983-1.063 |
| p-value | 0.293 | 0.231 | 0.21 | 0.311 | 0.279 |
| **LAG 6** | | | | | |
| OR | 1.053 | 1.08 | 1.009 | 0.977 | 0.979 |
| 95% CI | 0.890-1.246 | 0.965-1.187 | 0.978-1.041 | 0.951-1.003 | 0.943-1.017 |
| p-value | 0.544 | 0.2 | 0.569 | 0.077 | 0.283 |
| **LAG 7** | | | | | |
| OR | 0.955 | 0.962 | 0.999 | 1.020 | 1.022 |
| 95% CI | 0.825-1.106 | 0.877-1.054 | 0.971-1.027 | 0.997-1.044 | 0.987-1.058 |
| p-value | 0.541 | 0.4 | 0.934 | 0.09 | 0.216 |

Abbreviations: NO_2_, nitrogen dioxide; PM_2.5_, particulate matter with a diameter of 2.5 μm or less; PM_10_ particulate matter with a diameter of 10 μm or less; SO_2_, sulfur dioxide; CO, carbon monoxide; OR, odds ratio; 95% CI, 95% Confidence Intervals.

Table A11. Associations between the exposure to short-term pollutants and ischemic stroke incidence in the winter.

| **Variables** | **PM2.5** | **PM10** | **NO2** | **SO2** | **CO** |
| --- | --- | --- | --- | --- | --- |
| **LAG 0** | | | | | |
| OR | 1.109 | 1.089 | 1.028 | 1.028 | 2.303 |
| 95% CI | 1.002-1.227 | 1.010-1.174 | 1.006-1.050 | 0.980-1.079 | 0.806-6.578 |
| p-value | 0.045 | 0.026 | 0.013 | 0.26 | 0.119 |
| **LAG 1** | | | | | |
| OR | 0.907 | 0.916 | 0.975 | 1.007 | 0.452 |
| 95% CI | 0.807-1.021 | 0.836-1.004 | 0.951-1.000 | 0.955-1.060 | 0.126-1.616 |
| p-value | 0.105 | 0.06 | 0.047 | 0.805 | 0.222 |
| **LAG 2** | | | | | |
| OR | 1.019 | 1.048 | 1.012 | 0.988 | 1.118 |
| 95% CI | 0.905-1.148 | 0.957-1.148 | 0.987-1.038 | 0.935-1.043 | 0.313-4.000 |
| p-value | 0.755 | 0.309 | 0.359 | 0.662 | 0.864 |
| **LAG 3** | | | | | |
| OR | 1.026 | 0.983 | 1.001 | 1.028 | 0.714 |
| 95% CI | 0.906-1.163 | 0.893-1.081 | 0.976-1.027 | 0.974-1.085 | 0.187-2.724 |
| p-value | 0.681 | 0.72 | 0.925 | 0.315 | 0.622 |
| **LAG 4** | | | | | |
| OR | 1.059 | 1.024 | 1.016 | 1.044 | 1.807 |
| 95% CI | 0.935-1.200 | 0.931-1.127 | 0.990-1.043 | 0.991-1.101 | 0.471-6.926 |
| p-value | 0.364 | 0.625 | 0.217 | 0.106 | 0.388 |
| **LAG 5** | | | | | |
| OR | 1.014 | 1.052 | 1.009 | 0.979 | 1.934 |
| 95% CI | 0.900-1.142 | 0.960-1.152 | 0.983-1.036 | 0.929-1.032 | 0.524-7.141 |
| p-value | 0.824 | 0.282 | 0.492 | 0.435 | 0.322 |
| **LAG 6** | | | | | |
| OR | 0.921 | 0.954 | 0.99 | 1.006 | 0.404 |
| 95% CI | 0.820-1.035 | 0.872-1.043 | 0.966-1.015 | 0.954-1.061 | 0.110-1.489 |
| p-value | 0.167 | 0.3 | 0.429 | 0.817 | 0.173 |
| **LAG 7** | | | | | |
| OR | 1.003 | 0.986 | 0.994 | 1.012 | 0.778 |
| 95% CI | 0.910-1.105 | 0.916-1.060 | 0.972-1.016 | 0.966-1.060 | 0.266-2.274 |
| p-value | 0.96 | 0.696 | 0.59 | 0.613 | 0.646 |

Abbreviations: NO_2_, nitrogen dioxide; PM_2.5_, particulate matter with a diameter of 2.5 μm or less; PM_10_ particulate matter with a diameter of 10 μm or less; SO_2_, sulfur dioxide; CO, carbon monoxide; OR, odds ratio; 95% CI, 95% Confidence Intervals.
